# Perinatal healthcare experiences of pregnant and parenting people with a history of substance use disorder: a qualitative study

**DOI:** 10.1186/s12884-025-07473-8

**Published:** 2025-03-28

**Authors:** Carol Y. Franco-Rowe, Angela E. Lee-Winn, Venice Ng Williams, Connie Lopez, Gregory J. Tung, Mandy A. Allison

**Affiliations:** 1 Prevention Research Center for Family and Child Health, Department of Pediatrics, University of Colorado Anschutz Medical Campus, 1890 North Revere Court Mailstop F443 Aurora, Aurora, CO 80045 USA; 2 Department of Epidemiology, Colorado School of Public Health, University of Colorado Anschutz Medical Campus, 13001 E. 17th Place, MS B119, Aurora, CO 80045 USA; 3 Department of Health Systems, Management & Policy, Colorado School of Public Health, University of Colorado Anschutz Medical Campus, 13001 E. 17th Place, MS B119, Aurora, CO 80045 USA

**Keywords:** Perinatal substance use, Substance use disorder, Opioid use disorder, Medications for opioid use disorder, Healthcare

## Abstract

**Background:**

Clinical guidelines in the United States (U.S.) recommend a patient-centered approach to healthcare for pregnant people with substance use disorders (SUD); however, pregnant people with SUD often describe experiencing stigmatization and shame when seeking prenatal care. We explored the perspectives of pregnant and parenting people engaged with SUD treatment regarding their experiences with healthcare providers during the perinatal period to improve guidance for patient-centered care.

**Materials and methods:**

Using an adapted phenomenological approach, we conducted in-depth interviews with 22 pregnant and parenting people recruited from inpatient or outpatient substance use treatment centers in the U.S. state of Colorado. We developed an interview guide to explore participants’ experiences during pregnancy, childbirth, and postpartum. We audio recorded, transcribed, and validated interviews for analyses. A codebook was developed using an iterative process. Three coders analyzed the data and synthesized data into thematic memos.

**Results:**

Participants reported challenges within the healthcare system, including barriers to receiving services, connection to or education on resources, challenges in and reasons for sharing their history of substance use with healthcare providers, provider reactions to this information, and the impact of providers’ response to knowing about their substance use history. Participants described shame regarding their substance use but also a strong desire to ensure the health of their infants. This desire motivated them to share their history of substance use with healthcare providers. When participants perceived nonjudgmental and empathetic responses, they reported feeling pride and empowerment. Participants who reported judgmental responses from providers stated that it made them less likely to share and engage with other healthcare providers in the future.

**Conclusion:**

The perspectives and experiences of people engaged in SUD treatment can inform the implementation of clinical guidelines for patient-centered care for pregnant and parenting people in perinatal healthcare settings. Learnings from this study addresses ongoing challenges to compassionate care during this critical window, leading to disengagement of patients. Support through connection of resources can be helpful for ongoing recovery. Recommendations are made to establish trust through transparency and non-judgmental care and to reinforce receipt of appropriate healthcare services.

## Introduction

Substance use disorder (SUD) in pregnancy is a national public health concern, impacting thousands of pregnant people and their infants in the United States (U.S.) each year [1, 2]. In recent years, for example, the U.S. has seen increases in Neonatal Abstinence Syndrome (NAS) with six per 1000 newborn hospital stays being diagnosed with NAS according to 2020 national data [3, 4]. During the COVID-19 pandemic, increases in fentanyl use were observed among pregnant people, resulting in lower birth weights and increased preterm births [5, 6]. Untreated SUD can have negative health consequences for both child and parent, making the perinatal period a critical time to address SUD [7–16]. Early identification of substance use in pregnancy can offer improved outcomes for both parent and child, including referrals to substance use treatment and targeted prenatal care [17, 18].

Clinical guidelines in the U.S. recommends provision of patient-centered care to pregnant people experiencing SUD, however, some pregnant and parenting people may feel reluctant to seek prenatal care due to punitive state-level policies, prior negative personal experiences with the healthcare system, and fears over child welfare involvement [10, 19]. Birthing parents’ fears of legal or criminal consequences, including having their infants removed from their custody, lead them to be less likely to engage in healthcare, which places themselves and their infants at risk for negative birth and postnatal outcomes [20–27].

Stigma towards people experiencing SUD, including negative healthcare provider attitudes and adverse patient experiences further adds to reluctance to seek appropriate and timely prenatal care [28–31]. Pregnant people with a history of SUD often experience stigma in healthcare settings and are viewed as unfit to parent or inadequate caregivers [29, 32–35]. Being characterized as unfit to parent, particularly from respected healthcare professionals, can result in legal action or removal of parental rights [29, 36]. Clinical guidelines from Substance Abuse and Mental Health Services Administration (SAMHSA) and the American College of Obstetricians and Gynecologists (ACOG) recommend providing individually-tailored, empathetic, and nonjudgmental care to pregnant people with SUD [1, 37, 38], while the American Psychiatric Association (APA) recommends non-discriminatory and nonpunitive approaches to support initiation or continuation of treatment and services for those experiencing SUD [28]. These guidelines are based on evidence that pregnant people are motivated to seek treatment and cease substance use during pregnancy, often due to their concern for their child’s health and desire for stability for their children and themselves [39, 40]. However, previous research suggests that many healthcare providers have negative attitudes towards patients with SUD that can result in suboptimal treatment and poor outcomes [41]. Even when receiving medications for opioid use disorder (MOUD), patients may experience negative responses from their providers and/or receive advice that differs from their treatment plan [42]. Furthermore, internal feelings of shame and guilt add to their reluctance to share about their substance use despite their desire for optimal health outcomes for themselves and their infants [22, 43].

Understanding the experiences of pregnant people with SUD is needed for the improvement of existing guideline implementation. Highlighting pregnant and parenting people’s voices during this critical period may guide providers to deliver more compassionate care and facilitate trusting relationships [1, 44, 45]. A nonjudgmental, empathetic, and trauma-informed approach to care can help establish therapeutic relationships and repair patients’ negative views towards healthcare providers, particularly after having experienced negative interactions. Continued positive interactions in a healthcare setting can encourage patients to seek additional resources, such as MOUD or mental health counseling. Therefore, this study aims to understand the impact of patient interactions with perinatal healthcare providers on treatment and health seeking behaviors. This study adds to existing literature highlighting the experiences of patients with SUD in the healthcare setting from the perspective of participants who are currently in treatment for substance use disorders. Participants provided recommendations for both parents and healthcare providers to optimize health-seeking behaviors during the perinatal period. Participant perspectives can inform healthcare system processes or state-level policies, such as care coordination and referrals to resources, to improve overall care.

## Materials and methods

### Study design and population

We used an adapted phenomenological approach to examine the perspectives and experiences of people aged 18 and over receiving treatment for SUD who were pregnant or within one year of childbirth regardless of whether they currently had custody of a child. An adapted phenomenological approach is particularly valuable for investigating the dynamics of patient-provider relationships in healthcare research. By using this method, we maintained flexibility in our techniques, combining semi-structured interview guides to steer our questioning while providing space for participants to reflect on their subjective experiences and express the meanings they found in those experiences [46]. The semi-structured and flexible interviews enabled participants to share what was most significant to them within the context of perinatal healthcare with a history of substance use. The thematic interview guide included topics to understand participants’ experiences during pregnancy, childbirth, and early parenting (see Table 1). We used broad lines of questioning to understand participants’ experiences within the healthcare system (such as receiving prenatal care or during delivery), their decisions to seek treatment for substance use, their experiences receiving treatment, and any challenges or barriers in these contexts. The interview questions were developed by a team of perinatal health researchers, including experts in services and programs for pregnant and parenting individuals with substance use disorders, as well as addiction specialists. To capture the true essence of participants’ experiences, we chose not to mention race or ethnicity unless participants explicitly shared this information. In line with phenomenological principles, our focus was on exploring the lived experiences and the meanings that emerged from them. Therefore, we did not specifically request this information unless it was relevant to their experience.

**Table 1:** Interview guide

Theme	Interview Questions and Probes
Background	Can you tell me about yourself? • Age • Job/school • Living arrangements • Initiation of substance use
Pregnancy and delivery	How did you find out that you were pregnant? • What has it been like? • Were there things that made your pregnancy easier or harder? • Has anyone been an important part of your pregnancy experience? • Do you think about the future for you and your baby? o What does that look like?
	What was your delivery experience like? • What was your time like in the hospital after the baby was born? • How was your pain managed during labor? • How was your pain managed after delivery? • What was the baby’s time like in the hospital after he/she was born?
	What were the first few days like at home with you and the baby? • How was the baby sleeping and feeding? • Did you have any visitors?
	What was like the first few weeks at home with the baby. • Were there things that made the first few weeks at home easier or harder?
	How did substance use influence your birth experience if at all?
Treatment	What kind of substance and other factors brought you into treatment?
	How did you decide to seek treatment for substance use?
	What has your experience been like at the treatment center?
	Have you been able to become sober? • Why/why not?
Suggestions and Recommendations	If you had a room full of doctors and nurses, what would you want them to know about your experience during pregnancy and delivery? What would you want other mothers to know who may be in a similar situation as yours?

### Setting and recruitment

We recruited participants from three SUD treatment centers in Colorado: one inpatient residential treatment facility for women and two outpatient comprehensive treatment program centers. The residential facility specifically served birthing people in treatment for SUD and allowed pregnant and parenting people with young children to reside in their facility for up to 90 days while receiving individual and group therapies. The outpatient centers provided individual and group therapy and administered MOUD, such as buprenorphine and methadone. We recruited participants by providing flyers at outpatient centers with a phone number to participate via phone interview with a researcher. Researchers also conducted drop-in interviews at the residential facility where participants could visit the interviewer in a private office to participate in the interview. The study was approved by the researchers’ local institutional review board. All participants provided verbal consent to research staff and demonstrated their understanding of the study’s purpose. Participants were compensated with a $50 gift card for their participation.

### Data collection and analysis

We conducted interviews between May and October 2019 with 22 participants in substance use treatment. Each participant was treated as a single unit of analysis. Three researchers conducted interviews in-person or by telephone, with an average of 36 min per interview. We continued to conduct interviews until we met data saturation. Our team concluded that data saturation was achieved when interviewers reached a consensus that no new information was emerging from the participant population. We agreed that the themes were recurrent and consistent among participants. Interviews were recorded via a digital audio recorder and transcribed by an external contractor. The research team validated transcripts to ensure transcription accuracy. All data were de-identified to ensure anonymity. An adapted phenomenological approach allows us to capture the core of participants’ experiences by identifying themes and patterns during analysis, while maintaining a balance between description and interpretation [46]. This technique is particularly useful in healthcare research, as it reveals the subjective experiences of participants and the emotional influences on their decision-making processes [46]. Three independent coders then conducted thematic analysis using data-driven, iterative processes, developed a coding scheme, and completed triple coding with 25% of the transcripts to ensure inter-coder consistency (kappa > = 0.6). We conducted coding consistency meetings to discuss code definitions and reconcile any differences in coding. After coding, we wrote memos to synthesize codes into categories and characterized broader themes among all participants. We presented memos to a committee of healthcare providers and substance use treatment experts to validate and reflect on findings. We used NVivo 12 for data management and analyses [47].

## Findings

Most study participants were multiparous parents (77%), with the remainder either currently pregnant with or parenting one child (23%). Most participants were receiving inpatient residential treatment (65%), had delivered their babies, and were postpartum (65%) at the time of the interview (see Table 2).

**Table 2:** Participant characteristics

Total Number of Participants		22
Total Number of Interviews		23*
	Total (*n*)	Percent
**Setting**	23*	
Inpatient residential treatment for women	15	65%
Outpatient comprehensive treatment center #1	6	26%
Outpatient comprehensive treatment center #2	2	9%
**Pregnancy Status**	23*	
Pregnant when interviewed	8	35%
Delivered/postpartum when interviewed	15	65%
**Parity**	**22**	
First-time pregnancy or birth (primiparous)	5	23%
Second (or subsequent) pregnancy or birth (multiparous)	17	77%
**Other Characteristics**	**22**	
Participating in medically assisted treatment	12	56%

*One participant was interviewed twice: once during pregnancy while receiving outpatient treatment, and a second time postpartum while in residential facility. The participant did not disclose her previous interview with another researcher, and the interviewer was unaware of it. In both interviews, she shared the same information with the researchers.

All participants discussed their healthcare experiences during pregnancy, delivery and/or postpartum, including interactions with perinatal healthcare providers during scheduled prenatal appointments, during delivery, postpartum visits, in the Neonatal Intensive Care Unit (NICU), and with first responders. Our analyses revealed five major themes: (1) perception of barriers to accessing care and/or sharing one’s current or history of substance use; (2) reasons for sharing their substance use; (3) healthcare experiences after sharing of substance use; (4) impact of provider response to sharing of substance use on mothers; and (5) recommendations for other mothers with SUD and providers who may encounter mothers with SUD.

### Perception of barriers to accessing care and/or sharing their substance use history

All participants described barriers to accessing care and/or sharing history of their substance use with their providers and family members that largely related to their emotions or fears related to their use. Participants were hesitant to access care and/or share their substance use history with family or healthcare providers because of feelings of guilt and shame as well as distrust towards the healthcare system based on their previous healthcare experiences. Many also feared judgment by healthcare professionals and the possibility of social services or law enforcement involvement that may result in losing custody of their children.

When participants described feeling guilt, embarrassment, or shame, they talked about these negative feelings in the context of exposing their infants to substances during pregnancy. For example, one participant in inpatient services described this feeling as, *“At first you feel so ashamed. So*,* just guilty. Like*,* how could you do this to a child?” (participant 11)*. A few participants feared that judgment and scrutiny for their substance use would result in a negative response or treatment by their healthcare providers. Specifically, they worried about receiving substandard care due to providers’ negative bias towards pregnant people who use substances. For example, another participant stated, *“Am I going to get judged when I get to the hospital and have to tell them that I am on methadone*,* and… what’s their response going to be?” (participant 19).*

Reluctance to share their substance use history also stemmed from specific fears of having social services involvement, such as Child Protective Services, or being criminally penalized for their prenatal substance use. Some participants admitted that they did not know the laws surrounding substance use during pregnancy and/or delivery and whether any legal action or child welfare investigations would take place. Other participants had previously lost custody of their children and described that these experiences contributed to their current fears of losing custody of their infant. Such fear of potential adverse consequences acted as a barrier to sharing their substance use history, particularly during their prenatal visits. For example, one pregnant mother in inpatient treatment expressed her fears about possible repercussions of sharing this information, *“Like what’s going to happen? Are they going to take me to jail? Are they going to take my baby?” (participant 5).*

Most participants described distrust of the healthcare system, healthcare staff, and social service providers, often because of previous negative experiences in a healthcare setting. Some participants reported distrust towards their provider because they felt their provider was not being open and transparent with them. This lack of transparency included not being forthcoming about the purpose and results of various medical tests and how test results would be used. A few participants expressed feeling skeptical about their provider’s intentions and felt there were other motives for requesting biological samples. These participants implied that transparency from their healthcare provider is important for making participants feel comfortable and that they are receiving appropriate care. One participant stated, for example, “*You see*,* you don’t trust anyone… You see someone with a badge of any kind on*,* like whether it’s a plastic badge*,* a nurse badge*,* or a key card*,* you*,* I automatically assume that they’re*,* they work for the government*,* or they work for an organization*,* or their motivation is not to help me” (participant 13).*

### Reasons for sharing their substance use history

Despite the emotions and challenges associated with accessing care and disclosing their substance use history, all participants ultimately chose to share this information with their healthcare providers. While all participants shared about recent circumstances leading them to treatment (e.g., currently pregnant or children removed from their care), many also shared about their substance use history in prior pregnancies. Several participants described feeling motivated to protect their infant by sharing their substance use history to their provider, despite any potential negative consequences. Ensuring that they received appropriate medical care for themselves during pregnancy and for their infants after delivery was the most important task for these participants. Most participants were aware of the negative health consequences of using substances during pregnancy and that their substance use could have negative impacts on their infants’ health, such as developmental delays and experiencing withdrawal symptoms. Often, these participants described feeling anxious that their infant would suffer the consequences of their SUD. A mother receiving outpatient services expressed this sentiment, saying *“At first*,* I wasn’t going to tell them*,* but as things were*,* I just thought…*,* ‘Oh my God*,* I better tell them because what if something’s wrong.’ I needed for them to have the right doctors around” (participant 10).*

Some participants desired to establish a collaborative relationship with their healthcare provider. The ability to gain information about their health, pregnancy, and infant’s potential for withdrawal were reasons for sharing about their substance use. These participants shared that being upfront about their substance use with their provider early on enabled more transparent interactions and allowed them to receive better education throughout their pregnancy. These participants described that their openness had resulted in more appropriate care, such as obtaining educational materials and any care plans that their provider had prepared regarding their pregnancy, substance use, and other health concerns. One participant said, “*Be honest*,* completely transparent with my doctors and everybody*,* and we’re just making sure everything’s good*,* and so far*,* everything’s been great and healthy. I’ve got a whole printout of… doctor’s notes on my last ultrasound*,* and everything’s perfect*,* so far*,* but you just never know…” (participant 2).*

### Healthcare experience after sharing of substance use history

Most participants described their perceptions of how their healthcare provider reacted and treated them after they shared their substance use in three major ways: positive, negative, and mixed. Some participants described positive provider responses as nonjudgmental and supportive of their substance use treatment. Participants described feeling encouraged and supported by healthcare providers who normalized their history of substance use by talking about how common it is, particularly the increase of opioid use resulting in the current overdose crisis. They used words like “sympathetic”, “nurturing”, “caring”, and “nonjudgmental” to describe the healthcare provider with whom they had a positive experience. Because participants felt a tremendous amount of guilt for their substance use during pregnancy, many expressed gratitude for having sympathetic providers who eased their distress and attributed having had a supportive provider for their easy and successful delivery. A first-time postpartum mother receiving outpatient services shared, *“The doctor that delivered my baby made me feel like*,* you know what*,* it’s going to be okay. What matters now is from this day forward… she encouraged me” (participant 10).*

On the other hand, a few participants recalled feeling mistreated and disrespected or had experienced derogatory remarks from their provider after sharing their history of substance use. These negative responses made them feel judged for having used substances and that their ability to parent was being questioned. Some also felt questioned about their decision to seek MOUD during pregnancy or after delivery. For example, they recalled being called words like “junkies” or “druggies”. A few participants described being “man-handled” or feeling dehumanized by their previous providers due to their history of substance use. Several participants described feeling like they were being looked down on by medical staff and that medical staff were disapproving of them or were rude to them because of their substance use history. One participant shared, “*I was trying to take care of her [her daughter]*,* and the social worker said the same thing. She said*,* you know*,* usually junkies aren’t just very good with their kids*,* especially during this point” (participant 22)*. Another participant stated, “*People being rude to me*,* so it was kind of weird at first…It’s just like*,* I… felt very judged*,* felt very much I was being looked at as just some junkie” (participant 24).*

Some participants shared mixed experiences with their providers after sharing their substance use history, largely dependent on whether the role of providers was primarily to take care of the birthing parent or the infant. For instance, one participant described her “mixed” experiences having had understanding providers during labor and delivery (birthing parent her-focused care) but had experienced judgment from providers in the neonatal intensive care unit (NICU; newborn-focused care), where her infant had to stay for three weeks due to withdrawal symptoms. The NICU providers’ judgmental attitudes toward her added to her hardship in dealing with the emotional distress she was experiencing due to her infant’s NICU admission. This single mother with an 8-week-old infant stated, *“The nurses in labor and delivery were a lot more understanding for the most part. It was in the NICU that I really noticed some of the judgments” (participant 17).*

### Impact of provider response to mothers’ sharing of substance use history

Participants who had experienced positive responses from their providers described feelings of pride and empowerment by making the decision to be honest about their history of substance use and to seek treatment. Sharing this information helped them feel more self-sufficient and were more likely to advocate for their and their infants’ health. These participants described how positive patient-provider relationships, including having nonjudgmental, open conversations, had resulted in encouraging experiences during their prenatal visits and/or delivery. These participants who felt trusted and supported by their providers felt that providers believe the participants were doing the best they can. Many of these participants also stated that they trusted that providers would do their best to serve and support them through their pregnancy. One participant recounted, “*So*,* and I told her [OB provider] that*,* like when I became pregnant*,* I was like*,* guess what! She’s like*,* ‘Oh please just tell me you haven’t been drinking*,*’ and I said*,* ‘I haven’t! You’d be so proud!’ So*,* I was completely honest with her. She knows that I am here at [treatment center]” (participant 11)*.

In contrast, participants who had negative provider experiences after their sharing of substance use felt shamed, greater distrust of healthcare providers, and greater discomfort in future sharing of their history and seeking healthcare. For example, a mother receiving inpatient services who had seven prior pregnancies, shared her reluctance to return to a healthcare provider due to a prior negative experience, *“[While] giving birth*,* so I felt like they man-handled me a lot… more so than a woman delivering that was a sober female… I didn’t bother going back because I didn’t like their treatment” (participant 6)*. Participants who were pregnant while using substances expressed feeling more shame by the negative remarks or actions by their providers. As a result, some participants were less likely to share their history of substance use to other healthcare providers in the future. A few other participants stated that they had stopped going to see their current provider and chose a different provider or delayed seeking medical care despite having health concerns. Some participants shared that they believe the negative treatment from healthcare providers can lead to depression and could potentially contribute to relapses.

### Experiences with care coordination and resources

Most participants shared about their needs for coordination of services, resources, and education, particularly those relating to substance use treatment. These participants shared that they needed and valued education about substance use treatment during pregnancy, information about infant development, access to substance use treatment, access to safe housing, and access to programs to prevent food insecurity. Several participants expressed gratitude for the resources they had received, and a few described the impact that receipt of these resources could have on their lives. They felt that guidance and support from case managers and social workers were helpful in accessing resources that they otherwise would not know existed or how to apply. One participant shared about the importance of community supports which is a critical element to help people with a history of substance use stay sober, “*I think that the community support is very important for women*,* especially women who’ve just had babies who are also just getting sober. I feel like community support is probably one of the best things they could have*,* because I*,* with all my other tries at sobriety*,* I’ve never felt this much support. And then I’m*,* they’re helping me find housing as well*,* because I have a case manager while I’m here so they’re helping with housing and like anything else that I’ve needed to get while I’ve been here [residential substance use treatment program]*,* they’ve helped me get that. I have TANF (temporary assistance for needy families) now. I have my food stamps. I’ve got WIC… it’s been nice having somebody like [case manager] guiding along that path*,* and going*,* oh do you need to do this and this? And like kind of bring you back to actual life and helped me realize the things I need to get done” (participant 5).*

Meanwhile, a few participants talked about struggling to get needed resources, particularly housing and substance use treatment. Challenges exist when healthcare providers are aware of a patient’s substance use but do not offer support or resources. A couple of participants reflected on previous pregnancies where tests indicated that they had used substances, but they were not provided the proper education on risks associated with prenatal substance use and how or where to seek treatment. One participant reflected on lack of care coordination at prenatal visits despite having a positive screening for substances, *“Why didn’t none of these professionals*,* kind of act as*,* you know*,* a case manager*,* ‘Well*,* hey*,* you know you can go to this place while you’re pregnant*,* so you don’t give another birth with a child hooked on crack cocaine.’ You know*,* none of these professionals said that. All they did was recorded it.”* Referring to the case manager, *“It would have been nice*,* because a lot of women do not know all the resources that are out there*,* and they’re kind of already feeling like in a hopeless situation” (participant 21).*

### Recommendations from participants

Participants who had been honest with their healthcare providers about their history of substance use stated that they would advise other participants to share their substance use despite feeling afraid or ashamed, as doing so will help them receive the care they need. A couple participants made clear that SUD transcends socioeconomic status, and that it could happen to anyone regardless of one’s background. They also advised that participants should not be ashamed “to speak up” because it could help them receive appropriate resources. One mother in inpatient treatment suggested, *“It’s important for you to be completely honest during your pregnancy because they can’t treat you properly*,* you know what I mean*,* if not*,* they don’t know everything*,* they can’t treat you properly” (participant 12)*.

Some participants also provided recommendations for healthcare providers who care for patients with SUD. These participants wished that their providers viewed them without judgment. They would like providers to be kind, empathetic, and supportive, and recognize that they are doing their best to be a good mother for their children despite their SUD. Participants recommended that providers create a safe space for participants with SUD and be free of criticism towards them. Several participants suggested that healthcare providers need to take the time to connect with their patients and emphasized the need to not shame or blame the mother for disclosing her history of substance use. For example, one mother recommended that providers should recognize that SUD is not a defining characteristic but rather a result of their circumstances. She suggested that providers should *“Understand better that we’re still people. You know that we’re not bad people*,* we’re just troubled. We’ve been dealt a bad hand maybe but not all of us are bad” (participant 13)*.

Many participants talked about how important their role as a mother is to them and wanted healthcare providers to acknowledge and appreciate that side of them. Many participants wanted providers to understand that their substance use was a result of a history of adversity and not a permanent or defining trait. One participant, stated, for example, “*I know that I’m a good mom*,* you know*,* and… I know that I take good care of my babies… but all the things like I felt I was being judged for… without them even really knowing me*,* like that part got to me” (participant 6).*

## Discussion

Our aim was to investigate the healthcare experiences and perspectives of participants receiving substance use treatment during the perinatal period. By doing so, we aimed to offer practical information and examples to support the implementation of treatment guidelines for perinatal healthcare providers. This would help promote health-seeking behaviors and foster trust in the healthcare system among women who may be using substances. While ACOG and SAMSHA guidelines were published in 2012 and 2018, respectively, the experiences described by our participants after publication of these guidelines suggest that healthcare providers have not consistently been able to implement them successfully. By shedding light on patients’ actual experiences during care, this study’s findings can guide efforts to support healthcare providers who care for pregnant and parenting individuals with SUD.

Participants in this qualitative study expressed feelings of shame and guilt about their substance use and worries of stigma and scrutiny which were barriers to sharing with their healthcare providers. These findings align with the existing literature on prenatal substance use that the shame, fear, and guilt of participants who use substances during pregnancy experience is a key barrier to seeking treatment [48–54]. Though previous research has identified structural barriers such as lack of transportation, long wait times for appointments, and lack of childcare to seeking treatment, these barriers were not discussed by participants in this study [55, 56]. Providers’ reactions and treatment of participants following sharing of their substance use had a direct influence on participants’ emotional stress and their treatment-seeking decisions. A few participants discussed feelings surrounding negative experiences with healthcare providers, including feeling shamed, judged, and stigmatized: similar to findings from other qualitative studies [22, 50, 57, 58]. Several studies have found that pregnant and parenting people have experienced disparaging comments and behaviors in the healthcare setting, including feeling that they and their babies were being treated poorly by NICU nurses [57, 59, 60]. Some participants in this study also recalled experiencing these types of encounters.

Other participants who had more positive experiences felt empowered to advocate for their health and recovery and developed more trusting relationships with their providers. Existing recommendations to encourage engagement in prenatal care and treatment for SUD include the use of non-stigmatizing language, such as federal policy transitioning to “people first” language, replacing phrases like “addicts” to “people who use drugs” [61]. Our findings suggest that guidelines could provide additional examples of people-first, non-stigmatizing language, or scripts that healthcare providers could adopt to improve empathetic and nonjudgemental communication. In addition, guidelines could provide suggestions for language that healthcare providers could use to acknowledge that substance use is common and to praise and encourage pregnant people with substance use disorder who have revealed their substance use and sought treatment. The National Harm Reduction Coalition, in collaboration with the Academy of Perinatal Harm Reduction, for example has provided recommendations for motivational interviewing that use non-stigmatizing language to facilitate conversations that are trauma-informed and nonjudgmental [62]. Finally, healthcare providers would benefit from more guidance regarding practical implementation of an approach that identifies a parent’s strengths in addition to their challenges. For example, a validated tool to assess strengths could be developed and implemented specifically for this population [63].

Participants’ anticipated punitive responses instead of therapeutic and public health responses to their sharing of substance use history was a major contributing factor in participants’ reluctance to access care. This is consistent with the literature that has examined the unintended consequences of punitive state policies on prenatal substance use [64–68]. Although national and international organizations recommend the provision of a nonpunitive approach to care to reduce barriers in accessing care, participants were concerned about how care providers would treat them [69]. Many participants did not know laws surrounding punitive actions that may be taken against them, or whether their children would be removed from their custody and were thus a deterrent to seeking care. Other studies have found these fears contribute to lack of sharing of SUD to healthcare providers or not obtaining prenatal care, pointing to a need for transparency by healthcare providers about what actions would take place to patients with SUD [22, 70]. This latter point was emphasized by participants in our study. Despite national organizations recognizing that lack of consent to testing, punitive approaches, and “test and report” policies can damage patient trust and prevent patients from seeking care, we found that participants still faced stigma and fear in clinical settings [69]. These findings indicate that healthcare providers require additional resources, such as training, education, and financial support to effectively implement the recommendations from ACOG, SAMHSA, and APA. This includes providing accurate education, counseling, and referrals for MOUD using empathetic, trauma-informed, and harm reduction approaches [31, 62, 71].

Participants in our study wanted information about the risks of substance use during pregnancy and believed that education and guidance in treatment would help them to deliver healthy babies. Providers can support patients by providing education on the risks of substance use during pregnancy and available treatments. Guidelines suggest that providers use shared decision-making with their patients [72]; however, healthcare providers may lack the knowledge, time, and tools to implement shared decision-making. A shared decision-making tool might be a useful augmentation to existing guidelines [73]. Some hospitals have implemented statewide perinatal quality collaboratives to improve screening, initiation, and adherence to MOUD treatment and counseling, while also reducing racial disparities screening and MOUD access [74]. Connecting patients to available resources such as housing and food programs was valuable to participants in their path to sobriety. Ensuring that basic needs are met with support from staff such as case managers and social workers can help patients focus on their treatment and plan for the arrival of their infant. Collaborative and holistic approaches to SUD care can help reduce barriers to appropriate care, including clinical training to mitigate bias and the facilitation of patient referrals [31, 75].

In line with previous research, pregnancy was a window of opportunity for the study participants to seek treatment, despite their fears, because of their increased motivation to be honest about their substance use history to protect the health of their infants [76]. Ensuring their infants receive proper medical care when needed was the main reason for sharing their substance use history with providers. Participants’ concerns about the well-being of their infants, such as fetal growth, development, and possible withdrawal symptoms outweighed their fears of potentially losing custody of their infants to social services, therefore losing the opportunity to parent their children. This finding is consistent with existing literature on pregnant participants’ willingness to participate in programs that aim to reduce or cease prenatal substance use to improve their health with the goal of having a healthy infant [4, 49, 77]. This finding further demonstrates the need during this critical window for establishing trust among pregnant people in the healthcare system, through transparency and nonjudgmental care to reinforce health-seeking behaviors. Healthcare providers and systems could use further guidance and support to address issues of transparency. For example, sample policies and procedures could be provided to clarify when a drug test should be ordered, to obtain explicit verbal and written consent prior to drug testing, and to provide clear communication to patients regarding with whom their information and test results will be shared.

This study has limitations, as our participants were a convenience sample of participants in inpatient or outpatient substance use treatment programs in one U.S. state who were willing to participate in an in-depth interview. As our main purpose was to assess the perspectives and experiences of participants in SUD treatment, we did not conduct interviews with healthcare providers and staff who provide care for participants with SUD. A future study exploring providers’ perception and experience will be valuable.

Despite these limitations, our findings make a valuable contribution to the literature by sharing the lived experiences of participants with SUD. Examples of these lived experiences and sample language or scripts could be integrated into guidelines for care of pregnant and parenting people with substance use disorder to improve healthcare providers’ understanding and adoption of recommended practices. These lived experiences demonstrate that healthcare providers and systems need additional education, financial and personnel resources, and tools to successfully implement the ACOG, SAMSHA, APA guidelines.

## Data Availability

The deidentified data used and/or analyzed during the current study are available from the corresponding author on reasonable request.

## Abbreviations

ACOG: American College of Obstetrics and Gynecologists
APA: American Psychiatric Association
COVID-19: Corona virus disease of 2019
MOUD: Medications for Opioid Use Disorder
NAS: Neonatal abstinence syndrome
NICU: Neonatal intensive care unit
SAMHSA: Substance Abuse and Mental Health Services Administration
SUD: Substance use disorder
TANF: Temporary Assistance for Needy Families
U.S.: United States
WIC: Special supplemental nutrition program for women, infants, and children
